# IL-33 regulates TNF-α dependent effects in synovial fibroblasts

**DOI:** 10.3892/ijmm.2012.883

**Published:** 2012-01-10

**Authors:** ELKE KUNISCH, SARITHA CHAKILAM, MUKTHESHWAR GANDESIRI, RAIMUND W. KINNE

**Affiliations:** 1 Experimental Rheumatology Unit, Department of Orthopedics, University Hospital Jena, D-07607 Eisenberg; 2 Experimental Tumor Pathology, Institute of Pathology, University of Erlangen-Nuremberg, D-91054 Erlangen, Germany

**Keywords:** fibroblasts, interleukin-33, TNF-α, inflammatory diseases

## Abstract

The recently described IL-33 acts as a pro-inflammatory cytokine, inducing the expression of multiple responses in the target cells. Although a nuclear localization of IL-33 has been described, its exact functional relevance is presently unknown. The present study was conducted to analyze the effects of IL-33 on the TNF-α induced synthesis of the pro-inflammatory mediators IL-6, IL-8, and monocyte chemotactic protein-1 (MCP-1) and the pro-destructive molecules matrix metalloproteinase-1 (MMP-1), MMP-3, and TIMP-1 of rheumatoid arthritis synovial fibroblast (RA-SFs) using RNA overexpression and silencing. TNF-α significantly induced IL-33 mRNA expression and protein synthesis in RA-SFs. TNF-α-induced IL-33 protein expression was mediated via p38 signaling. Immunohistochemistry for IL-33 clearly showed that nuclear translocation of IL-33 was induced in TNF-α stimulated RA-SFs. IL-33 overexpression enhanced TNF-α-induced pro-inflammatory and pro-destructive functions in RA-SFs. IL-33 silencing significantly downregulated TNF-α-induced pro-inflammatory functions, whereas TNF-α-induced pro-destructive functions were less influenced by IL-33 silencing. This study identifies IL-33 as a critical regulator/enhancer of TNF-α-induced functions in RA-SFs, pointing to a central role of this cytokine in the perpetuation of pro-inflammatory and pro-destructive processes in rheumatoid arthritis (RA) and other inflammatory and degenerative diseases.

## Introduction

The 31 kDa protein IL-33 belongs to the IL-1 family of cytokines (1) and was first described as a nuclear factor produced by high endothelial venule cells (NF-HEV) (2). Precursor IL-33 seems to function as a regulator/repressor of nuclear transcription (3). This nuclear function is associated with the homeodomain-like helix-turn-helix motif in the N-terminal part of IL-33, which mediates DNA binding. A transcriptional regulatory function of precursor IL-33 is further supported by the nuclear localization of IL-33 in HUVECs and IL-33-overexpressing cells, but also in LPS-stimulated murine astrocytes (2–5). After secretion, mature IL-33 (18 kD C-terminal fragment) acts as a pro-inflammatory cytokine via a receptor complex of ST2 and IL-1 receptor accessory protein (IL-1RacP). As a pro-inflammatory cytokine, mature IL-33 induces the production of TH_2_ cytokines by TH_2_-cells and the secretion of numerous cytokines by human and murine mast cells (6,7). The biological effects of IL-33/ST2 are mediated by activation of the p38, ERK, JNK, and NF-κB signaling pathways (1,8). However, it has been recently shown that processing of precursor IL-33 results in an inactivation, rather than an activation of IL-33 (9). In addition, precursor IL-33 activates NF-κB and induces IL-6 synthesis. Therefore, it has been proposed that IL-33 functions as an endogenous danger signal (alarmin) to alert cells of the innate immune system to tissue damage during trauma or infection (9,10).

The IL-33/ST2 system is involved in numerous diseases and pathological conditions, e.g., fibroproliferative and cardiovascular diseases, asthma, and rheumatoid arthritis (RA) (8). An important role of IL-33 in the pathogenesis of RA is suggested by studies in the animal model of murine collagen-induced arthritis (11–14). Treatment of diseased animals with soluble ST2 fusion protein or a blocking anti-ST2 receptor reduced the disease severity compared to non-treated animals (e.g., reduction in synovial cellular infiltration, synovial hyperplasia, joint erosion and serum levels of pro-inflammatory cytokines); conversely, injection of IL-33 enhanced the signs of murine-collagen induced arthritis. Regarding human arthritis, IL-33 levels were elevated in the sera and synovial fluid of RA-patients and showed a positive correlation with the disease activity (15). IL-33 protein and also its receptor ST2 were detected in the lining layer and sublining of synovial membrane of RA patients (12). In addition to the strong expression of IL-33 in endothelial cells, fibroblasts and mononuclear inflammatory cells were identified as a potential source of IL-33 in the inflamed synovial membrane of RA patients (3,13). IL-33 mRNA and protein expression was induced in RA synovial fibroblasts (RA-SFs) following TNF-α/IL-1β stimulation and IL-33 protein was mainly detected in the nucleus of RA-SFs (12,13).

In addition to the role of mature IL-33 in the progression of RA (14,16), the nuclear localization of pro-IL-33 in IL-1β/TNF-α stimulated cells may point to a regulatory function inside the cells, as it has been described for the other IL-1 family members IL-1α and IL-1F7b (17,18). Therefore, the present study sought to analyze the involvement of IL-33 in TNF-α-induced pro-inflammatory or pro-destructive effector functions of RA-SFs.

## Materials and methods

### Patients, tissue digestion and cell culture

Synovial tissue from RA-patients fulfilling the ARA criteria was obtained during open joint replacement/arthroscopic synovectomy from the Clinic of Orthopedics, Eisenberg, Germany (19). The study was approved by the Ethics Committee of the University of Jena, Germany, and patient informed consent was obtained.

RA synovial samples were digested, subsequently cultured for 7 days, and RA-SFs negatively isolated as previously described (20,21). RA-SFs were cultured in the virtual absence of contaminating non-adherent cells and macrophages. Third-passage cells were used for all the experiments. Mycoplasma contamination of the cells was excluded by 4′-6-diamidino-2-phenylindole (DAPI) staining.

The stimulation of the cells with different concentrations of TNF-α (0.1 to 10.0 ng/ml; R&D Systems, Wiesbaden, Germany) was performed in DMEM/0.2% lactalbumin hydrolysate. For analysis of signal transduction pathways, cells were preincubated for 45 min with inhibitors of p38 MAPK (SB203580, 1 μM; Jena Biosciences, Jena, Germany), ERK (U0126, 1 μM, Axxora, Lörrach, Germany), JNK (SP600125, 20 μM, Jena Biosciences), NFκB (IκBK inhibitor peptide, cell-permeable, 50 μg/ml; Calbiochem, VWR, Darmstadt, Germany) (22), PKA (PKA inhibitor fragment 14–22, myristoylated trifluoroacetate salt, 10 μM, Sigma, Deisenhofen, Germany) (23) or PI3-kinase (wortmannin, 1 μM, Axxora), followed by TNF-α stimulation for 24 h. For analysis of the influence of different cytokines/growth factors on IL-33 synthesis, RA-SFs were stimulated with TNF-α, IL-18, PDGF-BB, TGF-β1 (10 ng/ml each), or IL-1β for 24 h (5 ng/ml; R&D Systems; Peprotech, London, UK). To analyze the influence of exogenous IL-33 on signal transduction and functional parameters of RA-SFs, cells were stimulated with 10 or 100 ng/ml recombinant human IL-33 (R&D Systems) for 15 min (signal transduction) or 24 h (protein secretion). Viability of the cells was assessed by ethidium bromide staining.

### Analysis of the IL-33 protein expression and signal transduction by Western blotting

Equal volumes of supernatants or 50 μg cellular protein were separated by 12% SDS-PAGE using a Laemmli buffer system as previously described (24). Primary anti-IL-33 antibody was obtained from R&D Systems. The band intensities were quantified using the software program Diana III luminescence imaging system (Raytest, Straubenhardt, Germany) and normalized to β-actin. Signal transduction in IL-33 and TNF-α stimulated RA-SFs was analyzed as previously described (25).

### Overexpression of IL-33 in RA-SFs

Overexpression of IL-33 in RA-SFs was performed using the ViraPower™ Lentiviral Expression System according to the manufacturer’s instructions (Invitrogen, Karlsruhe, Germany). In brief, the coding sequence of IL-33 was cloned into the plenty6/V5 vector. For producing lentivirus, 296FT cells were transfected with the ViraPower™ packaging mix, the pLenti IL-33 expression plasmid, or the empty pLenti expression plasmid using Lipofectamine™ 2000 and Opti-MEM^®^ I medium. After 24 h, the medium was changed to complete culture medium according to the manufacturer’s instructions (Invitrogen). Virus-containing supernatants were collected 72 h post-transfection, centrifuged to remove cell debris, and stored in aliquots at −80°C. For IL-33 overexpression, RA-SFs were transduced with the virus-containing supernatant in the presence of Polybrene™ (6 μg/ml; Sigma, Deisenhofen, Germany). After 24 h incubation, the virus-containing medium was replaced by DMEM/10% FCS and blasticidin for selection resulting in cells of passage 5. Overexpression of IL-33 was analyzed by RT-PCR and ELISA. As a control, RA-SFs were transduced with empty pLenty6/V5 vector.

### Silencing of IL-33 in RA-SFs

Silencing of IL-33 in RA-SFs was performed by the siRNA technique using reverse transfection according to the manufacturer’s instructions (Invitrogen). In brief, Stealth™ RNAi and Lipofectamine™ RNAiMAX (Invitrogen) were mixed in Opti-MEM^®^ I (Invitrogen) and incubated for 20 min at room temperature to allow complex formation. Subsequently, cells suspended in DMEM and 10% FCS without antibiotics were added and incubated for 24 h. Thereafter, cells were stimulated with 10 ng/ml TNF-α in DMEM and 0.2% lactalbumin hydrolysate for 24 h. Supernatants of the cells were collected for the analysis of cytokine and protease secretion. Cells were washed twice with ice-cold PBS and subsequently lysed in buffer for RNA-isolation (Macherey Nagel, Düren, Germany) or ice-cold buffer for protein extraction (50 mM Tris, 150 mM NaCl, EDTA, pH 7.4, containing 100 mM NP-40, 1 mM phenylmethylsulphonylfluoride, 1 mM Na_3_VO_4_, 2 μg/ml aprotinin, 2 μg/ml pepstatin, and 2 μg/ml leupeptin). For the analysis of IL-33 silencing, 35 μg of cellular protein were separated by 15% SDS-PAGE using a Laemmli buffer system. Transfection efficiency was analyzed using 10 nM Block-iT™ AlexaFluor^®^ Red Fluorescent Oligo (Invitrogen).

### Immunohistochemistry for IL-33 in RA-SFs

For IL-33-immunohistochemistry in RA-SFs, 0.4×10^5^ cells/well (8-chamber slides) were allowed to adhere for 24 h, stimulated for 24 h with 10 ng/ml TNF-α, 5 ng/ml IL-1β, 10 ng/ml IL-18, 10 ng/ml PDGF-BB, or 10 ng/ml TGF-β1 in DMEM with 0.2% lactalbumin hydrolysate, followed by fixation with 3.7% paraformaldehyde in PBS for 15 min at room temperature (RT) and by neutralization with 50 mM NH_4_Cl in PBS for 5 min at RT. Fixed cells were permeabilized with PBS/0.1% Triton X-100 for 5 min at RT. To inactivate endogenous peroxidase, cells were incubated with 0.03% H_2_O_2_/PBS for 20 min. Thereafter, cells were incubated with primary antibody (clone Nessy-1, Axxora) in PBS/1% bovine serum albumin for 2 h at RT, washed 3 times for 5 min each with PBS/1% BSA, and incubated with horseradish-peroxidase-coupled goat anti-rabbit IgG (Dako, Hamburg, Germany) in PBS/1% BSA for 1 h at room temperature. Cells were washed thoroughly with PBS and stained with DAB.

### Analysis of IL-33 mRNA expression and functional parameters by RT-PCR

Total RNA was isolated from RA-SFs and reverse-transcribed as previously described (26,27). mRNA expression for IL-33, IL-6, IL-8, MCP-1, MMP-1, MMP-3, TIMP-1, and the house-keeping gene aldolase was analyzed by real-time PCR using a RealPlex^®^ PCR machine (Eppendorf, Hamburg, Germany; (26,27). The primer pairs and PCR conditions are presented in Table I. The relative concentrations of IL-33, IL-6, IL-8, MCP-1, MMP-1, MMP-3, and TIMP-1 mRNA in each sample were calculated by the RealPlex^®^ software using an external standard curve. Product specificity of the real-time PCR was confirmed by: i) melting curve analysis; ii) agarose gel electrophoresis; and iii) initial cycle sequencing of the PCR products.

### Analysis of functional parameters in RA-SFs by ELISA

Proliferation was assessed by BrdU incorporation using a commercially available cell proliferation ELISA (Roche) as previously described (25). Human IL-6, IL-8, MCP-1, TIMP-1, and PGE_2_ were measured in diluted cell culture supernatants using commercially available ELISA kits (OptEIA™, BD Biosciences, Heidelberg, Germany; R&D Systems; GE Healthcare, Freiburg, Germany). Human MMP-1 and MMP-3 were measured in diluted cell culture supernatants as previously described (27). The resulting colour was read at 450 nm in microtiter plates spectrophotometer (Fluostar Optima, BMG LABTECH GmbH, Ortenberg, Germany).

### Statistical analysis

Data are presented as means ± standard error of the mean (SEM). The non-parametric Mann-Whitney U-test was applied to analyze differences between controls and individual stimuli, or among different stimuli (software program SPSS 10.0^TM^; SPSS Inc., Chicago, IL, USA). Significant differences were accepted for P≤0.05.

## Results

### Influence of TNF-α on the expression of IL-33 mRNA and protein in RA-SFs

**Figure 1:**
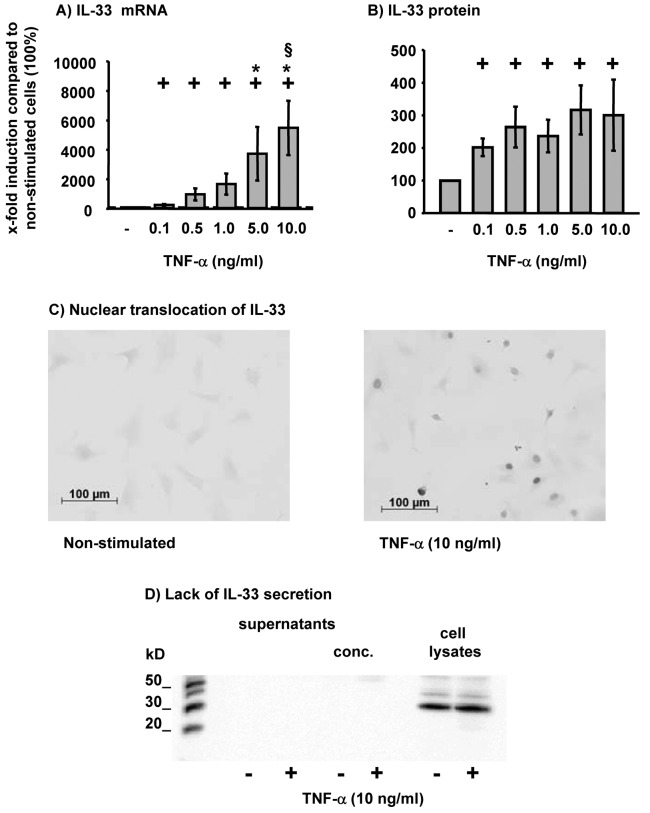
Influence of TNF-α on IL-33 mRNA and protein expression in RA-SFs and cellular localization of IL-33 in TNF-α stimulated RA-SFs. (A and B) RA-SFs (n=5) were stimulated with TNF-α (0.1, 0.5, 1.0, 5.0 or 10.0 ng/ml). The mRNA expression of IL-33 was analyzed by (A) RT-PCR, the protein expression by (B) ELISA. RA-SFs (n=4) were stimulated for 24 h with 10 ng/ml TNF-α. (C) Thereafter, IL-33 was detected in the cells by immunohistochemistry using a specific antibody against IL-33 or (D) in supernatants and cell lysates by Western blotting; conc, concentrated supernatants. Representative results are shown in (C and D). ^+^P≤0.05 vs.control;^*^P≤0.05 vs. 0.1 ng/ml TNF-α; ^§^P≤0.05 vs. 0.5 ng/ml TNF-α by the Mann-Whitney U-test. Bars indicate means ± SEM vs. control.

TNF-α significantly and dose-dependently induced the expression of IL-33 mRNA in RA-SFs (Fig. 1A). The expression of IL-33 was significantly induced starting at the lowest concentration of TNF-α (0.1 ng/ml; Fig. 1A) and further augmented by higher concentrations thereof (plateau at 5.0 or 10 ng/ml TNF-α; Fig. 1A). TNF-α also significantly induced IL-33 protein in RA-SFs (Fig. 1B); the production of IL-33 reached a plateau at the lowest concentration of TNF-α (0.1 ng/ml; Fig. 1B).

Non-stimulated RA-SFs showed a weak staining for IL-33 (Fig. 1C). However, following stimulation with TNF-α RA-SFs showed a strong positive staining predominantly localized in the nucleus (Fig. 1C). To analyze whether TNF-α-induced IL-33 protein is secreted, IL-33 protein was analyzed in the supernatants and cell lysates of RA-SFs following TNF-α stimulation. It was clearly shown that IL-33 was not secreted by RA-SFs, but remained exclusively in the cell lysates (Fig. 1D).

**Figure 2:**
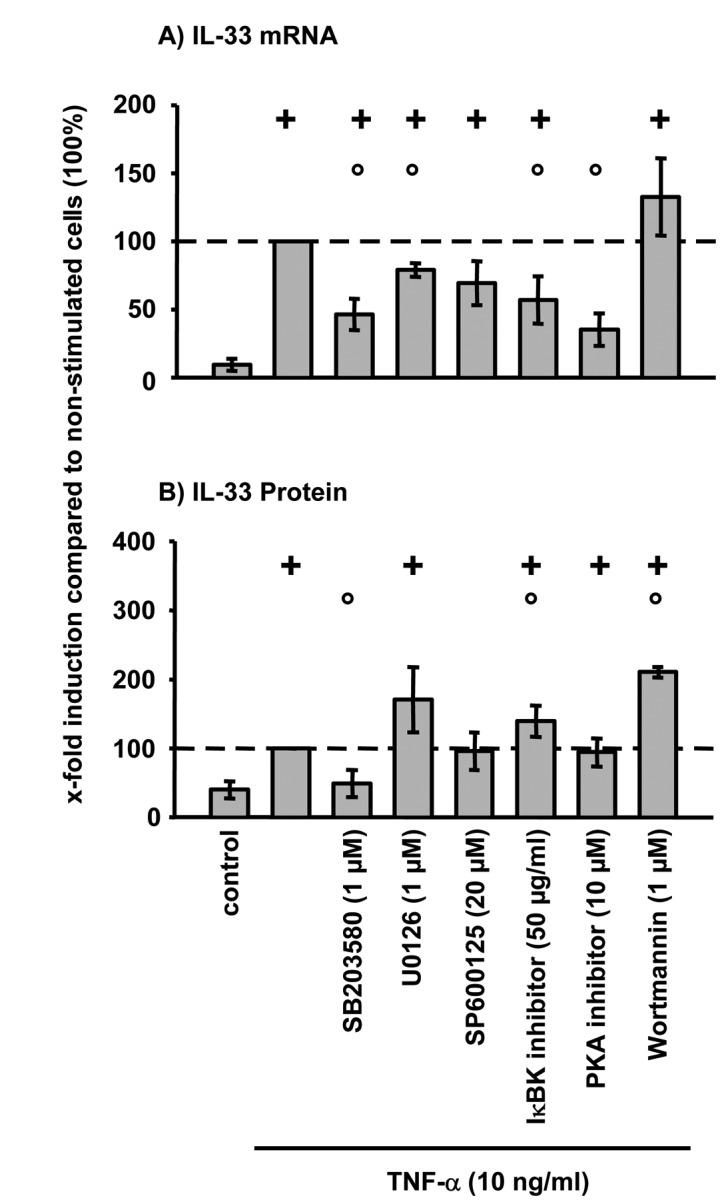
Signal transduction pathways involved in the TNF-α induced IL-33 expression in RA-SFs. RA-SFs (mRNA n=5; protein n=3) were preincubated for 45 min with the individual inhibitors as indicated followed by stimulation with 10 ng/ml TNF-α for 24 h. The mRNA expression of IL-33 was analyzed by RT-PCR (A) and protein expression by ELISA (B). ^+^P≤0.05 vs. control; °P≤0.05 vs. 10 ng/ml TNF-α without inhibitors by the Mann-Whitney U-test. Bars indicate means ± SEM.

TNF-α-induced IL-33 mRNA expression in RA-SFs was significantly reduced by inhibition of p38 MAPK (SB203580), ERK (U0126), NFκB (IκBK inhibitor peptide), and PKA (PKA inhibitor fragment 14–22) (Fig. 2A). Inhibition of JNK (SP600125) and PI3-kinase (wortmannin) had no significant influence on the TNF-α-induced IL-33 mRNA expression. However, at the protein level inhibition of p38 significantly reduced TNF-α-induced IL-33 (Fig. 2B). In contrast, inhibition of the NFκB pathway and PI3-kinase significantly induced IL-33 protein in RA-SFs. No significant viability differences were observed between non-stimulated cells (control) and TNF-α-stimulated cells with/without inhibitors (viability in all cases: >97%; data not shown).

### Effects of IL-33 overexpression on the TNF-α-induced functions of RA-SFs

**Figure 3:**
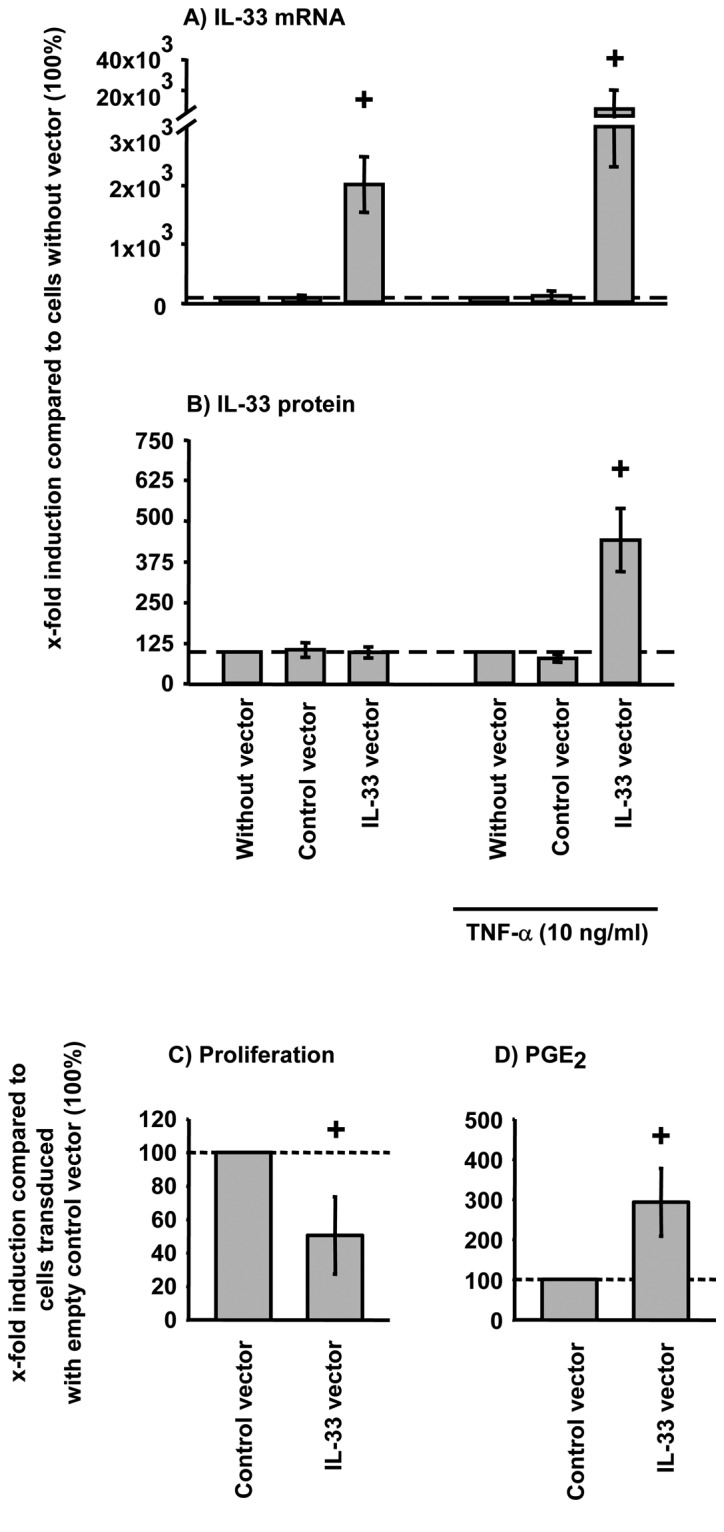
Lentiviral overexpression of IL-33 and influence of IL-33 overexpression on proliferation and PGE_2_ secretion in TNF-α-stimulated RA-SFs. RA-SFs (n=3) were transduced with empty plenty6/V5 vector or IL-33 plenty6/V5 vector and selected with blasticidin. IL-33 overexpression was analyzed following TNF-α stimulation using (A) RT-PCR or (B) ELISA. Proliferation of the cells was assessed by BrdU incorporation (C) and PGE_2_ secretion by ELISA (D). ^+^P≤0.05 Mann-Whitney U-test vs. control (cells without vector). Bars indicate means ± SEM.

Lentiviral overexpression of IL-33 mRNA significantly augmented the TNF-α induced IL-33 mRNA and protein expression in RA-SFs (Fig. 3A and B).

TNF-α significantly downregulated the proliferation of RA-SFs (data not shown) Overexpression of IL-33 further augmented the TNF-α induced downregulation of RA-SFs proliferation (Fig. 3C). The TNF-α-induced PGE_2_ secretion in RA-SFs, in contrast, is further enhanced by IL-33 overexpression (Fig. 3D).

**Figure 4:**
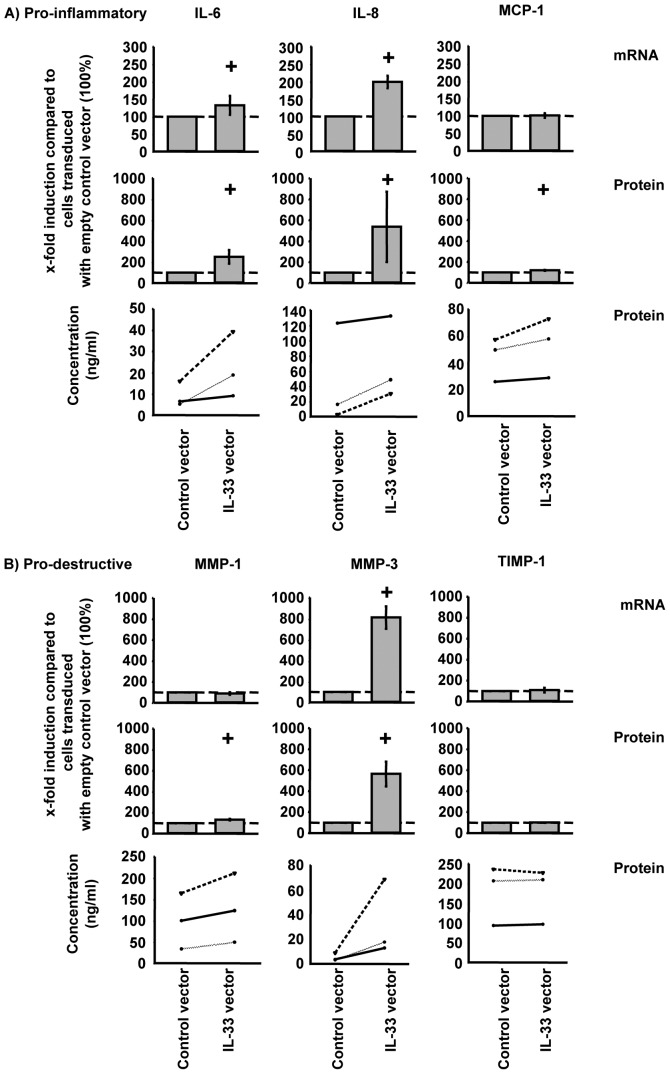
Effects of IL-33 overexpression on the mRNA and protein expression of pro-inflammatory and pro-destructive mediators in TNF-α stimulated RA-SFs. RA-SFs (n=3) were stimulated with TNF-α (10 ng/ml) for 24 h. mRNA and protein expression of (A) the pro-inflammatory mediators IL-6, IL-8, and MCP-1, and (B) the pro-destructive mediators MMP-1, MMP-3, and TIMP-1 were analyzed by real-time PCR or ELISA. ^+^P≤0.05 Mann-Whitney U-test vs. control vector. Bars indicate means ± SEM.

At the mRNA level, overexpression of IL-33 significantly increased TNF-α-induced IL-6, IL-8, and MMP-3 in RA-SFs (Fig. 4; shown as relative and absolute values). However, at the protein level, overexpression of IL-33 significantly induced the secretion of IL-6, IL-8, MCP-1, MMP-1, and MMP-3 (Fig. 4). Overexpression of IL-33 did not significantly influence TIMP-1 expression at the mRNA or protein levels (Fig. 4B).

### Effects of IL-33 silencing on the TNF-α-induced functions of RA-SFs

**Figure 5:**
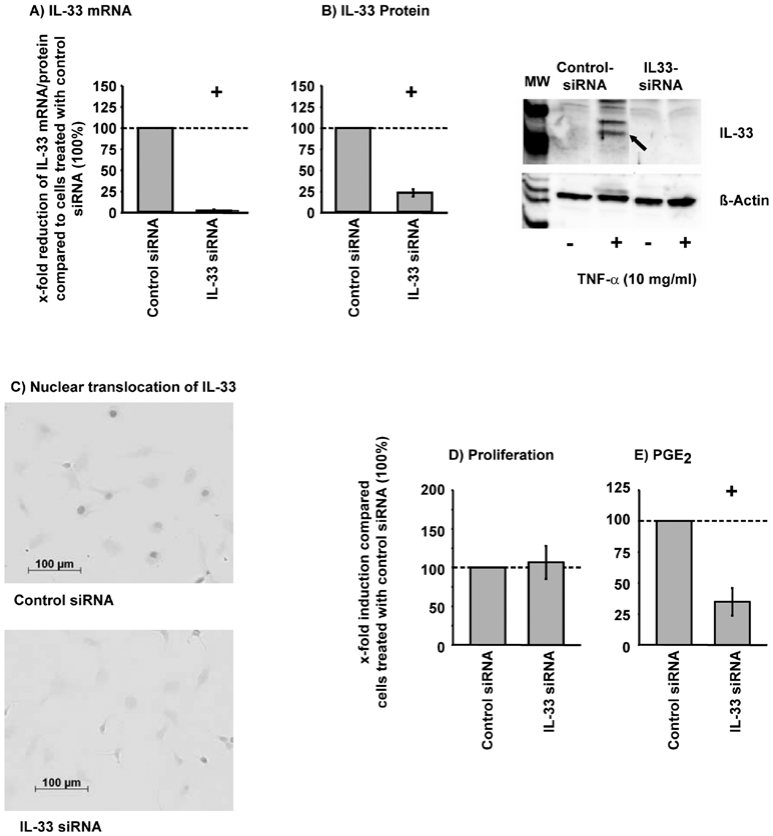
Silencing of IL-33 and influence of IL-33 silencing on proliferation and PGE_2_ secretion in TNF-α-stimulated RA-SFs. RA-SFs (mRNA n=4; protein n=3) were preincubated for 24 h with 10 nM control siRNA or specific IL-33 siRNA followed by TNF-α stimulation (10 ng/ml) for 24 h. IL-33 expression was analyzed by real-time PCR (A) and Western blotting (B) or by immunohistochemistry using a specific IL-33 antibody (B; in Fig. B and C a representative result is shown). Proliferation of the cells was assessed by BrdU incorporation (D) and PGE_2_ secretion by ELISA (E). ^+^P≤0.05 Mann-Whitney U-test vs. control siRNA. Bars indicate means ± SEM.

Silencing of IL-33 significantly reduced the TNF-α-induced IL-33 mRNA and protein expression in RA-SFs (Fig. 5A and B). Transfection efficiency was greater than 85% using Block-iT™ AlexaFluor^®^ Red Fluorescent Oligo (85.4% ±2.7 positive cells). In addition, immunohistochemical analysis of IL-33 siRNA showed that IL-33 siRNA abolished the TNF-α-induced nuclear translocation of IL-33 (Fig. 5C). The viability of the cells was not significantly influenced by silencing of IL-33 (viability, >97%; data not shown). The TNF-α-regulated proliferation of RA-SFs was not significantly influenced by IL-33 silencing (Fig. 5D). In contrast, silencing of IL-33 significantly reduced the TNF-α-induced PGE_2_ secretion (Fig. 5E).

**Figure 6:**
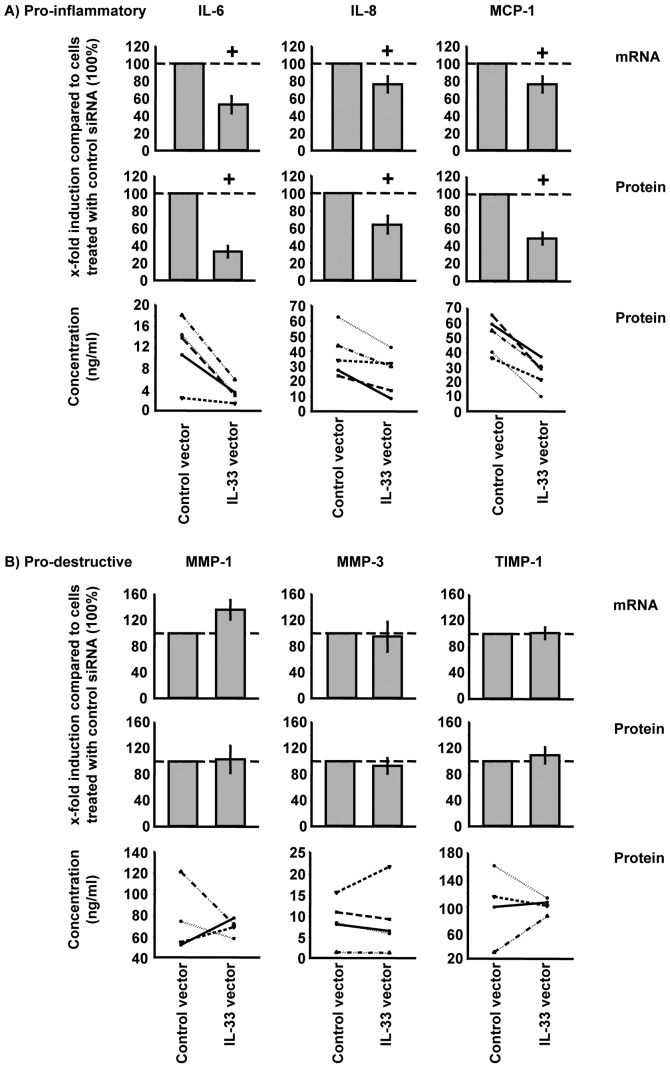
Effects of IL-33 silencing on the mRNA and protein expression of pro-inflammatory and pro-destructive mediators in TNF-α stimulated RA-SFs. RA-SFs (n=5) were preincubated for 24 h with 10 nM control siRNA or specific IL-33 siRNA followed by TNF-α stimulation (10 ng/ml) for 24 h. mRNA and protein expression of (A) the pro-inflammatory mediators IL-6, IL-8 and MCP-1, and (B) the pro-destructive mediators MMP-1, MMP-3, and TIMP-1 were analyzed by real-time PCR or ELISA. ^+^P≤0.05 Mann-Whitney U-test vs. control siRNA. Bars indicate means ± SEM.

Silencing of IL-33 significantly reduced the TNF-α induced synthesis of IL-6, IL-8, and MCP-1 in RA-SFs at the mRNA and protein levels (Fig. 6A). In contrast to the pro-inflammatory mediators, the synthesis of the pro-destructive mediators was not significantly influenced by IL-33 silencing in RA-SFs (Fig. 6B).

### Influence of different cytokines and growth factors on the IL-33 protein expression in RA-SFs

**Figure 7:**
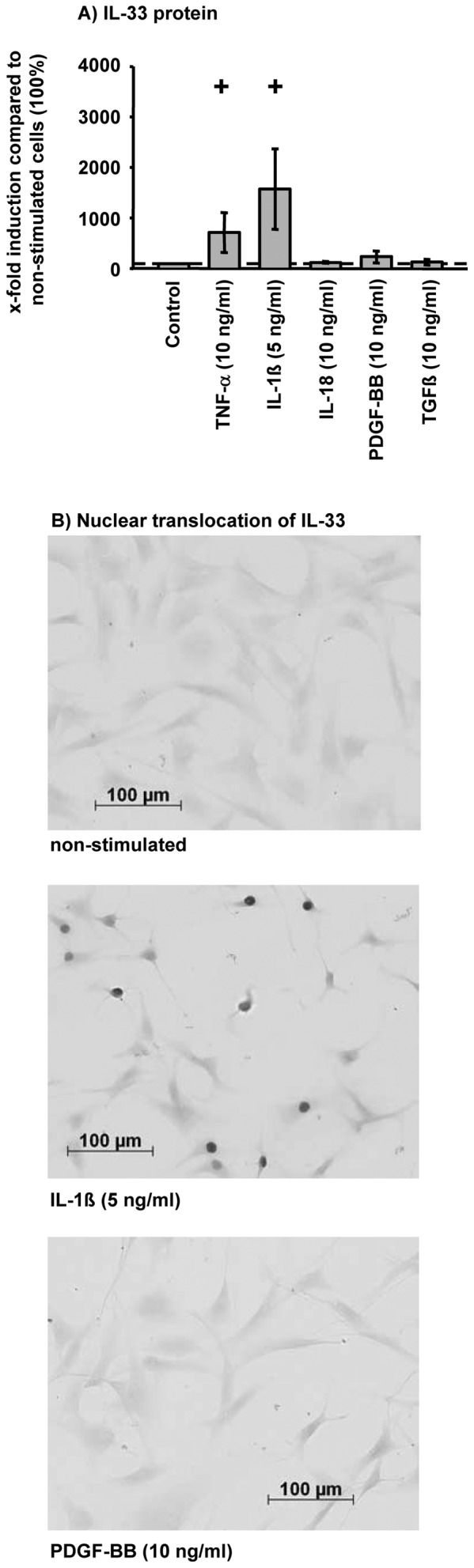
Influence of selected cytokines and growth factors on the IL-33 synthesis in RA-SFs. RA-SFs (n=4) were stimulated for 24 h with TNF-α, IL-1β, IL-18, PDGF-BB or TGF-1β (concentrations as indicated in the figure). Synthesis of IL-33 in the cell lysates was analyzed by ELISA (A) or in cells by immunohistochemistry using a specific antibody against IL-33. (B) Representative results are shown. ^+^P≤0.05 Mann-Whitney U-test vs. control. Bars indicate means ± SEM.

In addition to TNF-α, the pro-inflammatory cytokine IL-1β significantly induced IL-33 synthesis (fold-induction compared to control cells: TNF-α, 7.1-fold; IL-1β, 15.7-fold; Fig. 7A). In contrast, the pro-inflammatory cytokine IL-18 or the growth factors PDGF-BB and TGF-β1 had no significant influence on IL-33 synthesis. In agreement with these data, nuclear translocation of IL-33 was solely observed in TNF-α or IL-1β-stimulated cells (Fig. 7B; results for IL-1β and PDGF-BB are shown).

### Influence of exogenous IL-33 on signal transduction and protein expression of pro-inflammatory and pro-destructive mediators in RA-SFs

**Figure 8:**
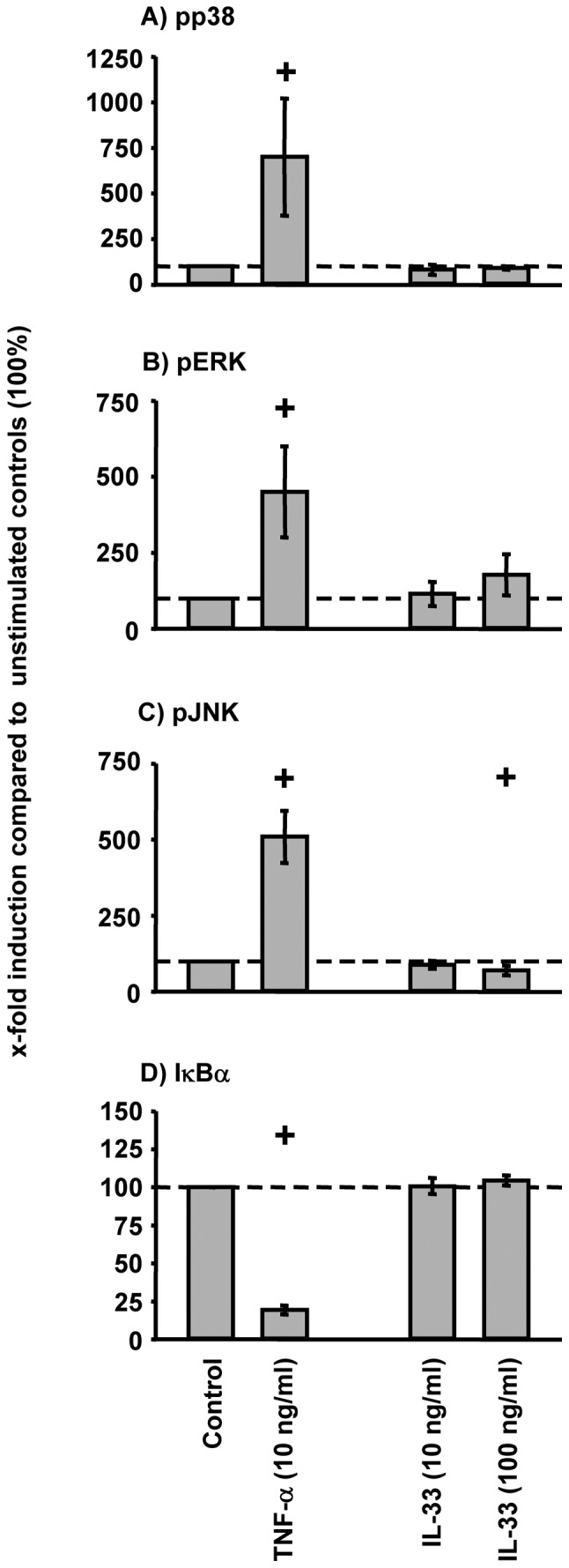
Influence of exogenous IL-33 on the signal transduction in RA-SFs. RA-SFs (n=3) were stimulated with IL-33 (10 or 100 ng/ml) or as a positive control with TNF-α (10 ng/ml) for 15 min. Activation of p38, JNK, ERK, and IκBα was analyzed by Western blotting. ^+^P≤0.05 Mann-Whitney U-test vs. control. Bars indicate means ± SEM.

**Figure 9:**
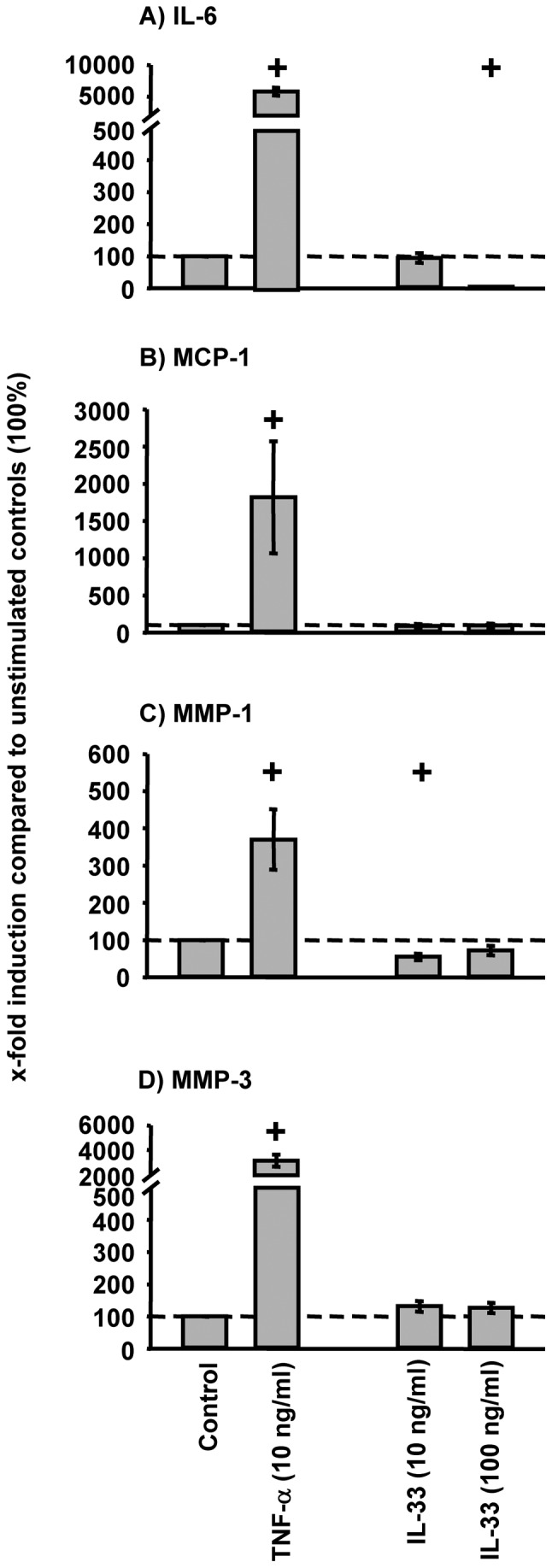
Influence of exogenous IL-33 on the protein expression of pro-inflammatory and pro-destructive mediators in RA-SFs. RA-SFs (n=4) were stimulated with IL-33 (10 or 100 ng/ml) or TNF-α (10 ng/ml) for 24 h. Protein expression of IL-6 (A), MCP-1 (B), MMP-1 (C), and MMP-3 (D) was analyzed by ELISA. ^+^P≤0.05 Mann-Whitney U-test vs. control siRNA. Bars indicate means ± SEM.

External IL-33 stimulation (10 or 100 ng/ml) of RA-SFs did not induce a significant increase in the phosphorylation of p38, ERK, or JNK (Fig. 8A-C). Also, no significant decrease of IκBα was observed following IL-33 stimulation of RA-SFs (Fig. 8D). Accordingly, IL-33 stimulation did not significantly induce the protein secretion of IL-6, MCP-1, MMP-1, and MMP-3 (Fig. 9). In all cases, TNF-α induced a significant activation of the analyzed signaling pathways and a significant increase of the secretion of pro-inflammatory and pro-destructive mediators (Figs. 8 and 9).

## Discussion

The present study demonstrates for the first time, that i) TNF-α induced IL-33 expression is regulated via p38; ii) the pro-inflammatory cytokines TNF-α and IL-1β are potent inducers of IL-33 synthesis; and iii) IL-33 is involved in the regulation of TNF-α-induced synthesis of pro-inflammatory and pro-destructive molecules. Therefore, IL-33 is a critical regulator of TNF-α-induced functions in RA-SFs, pointing to a central role of this cytokine in the perpetuation of pro-inflammatory and pro-destructive processes in RA and other inflammatory and degenerative diseases.

In agreement with previously published data, the present study shows that IL-33 mRNA/protein expression is induced in RA-SFs following TNF-α stimulation (12,13). Interestingly, IL-33 was only detected in the nucleus of TNF-α-stimulated RA-SFs, but not or only barely in the supernatant of stimulated RA-SFs [present publication, unpublished data and (13)]. This nuclear localization has also been observed in HUVECs and glia cells (2,4,5) and supports the proposed function of IL-33 as a transcriptional regulator (3). Nuclear translocation of IL-33 following TNF-α/IL-1β stimulation of RA-SFs therefore indicates that IL-33 may function as a transcriptional regulator rather than as an external cytokine. This view is further supported by the observation that the long signaling ST2 receptor is not expressed on the cells (13). In addition, RA-SFs do not respond to IL-33 stimulation with an activation of signal transduction molecules (p38, Erk, JNK, and NF-κB) or IL-6, MCP-1, MMP-1, and MMP-3 expression (present publication; (13). The incapability of RA-SFs to secrete mature IL-33 may be based on an inability of the cells to efficiently cleave pro-IL-33. Indeed, IL-1α, another member of the IL-1 family sharing several properties with IL-33, is only cleaved and secreted by monocytes and macrophage cell lines, but not by fibroblast cell lines (28,29). In addition, only detergent-damaged RA-SFs release the 30 kDa IL-33 precursor into the supernatant (15) further supporting the view that IL-33 is not actively secreted by RA-SFs.

Currently nothing is known about the signaling pathways involved in the TNF-α-induced IL-33 synthesis in RA-SFs. The present study shows for the first time that TNF-α-induced IL-33 mRNA expression is mainly regulated via p38, underlining its central pro-inflammatory role and identifying IL-33 as a new potential target for anti-rheumatic therapy with inhibitors of this signaling pathway.

IL-33 synthesis is differentially induced by cytokines and growth factors in RA-SFs. The pro-inflammatory cytokines TNF-α and, in particular, IL-1β strongly induce IL-33 synthesis. In contrast, the pro-inflammatory cytokine IL-18 or the growth factors PDGF-BB and TGF-β1 did not significantly stimulate IL-33 synthesis. In addition, a nuclear localization of IL-33 was only observed in IL-1β or TNF-α-stimulated cells, further emphasizing the prominent role of these 2 cytokines in regulating the expression/functional effects of IL-33. The induction of IL-33 by IL-1β and TNF-α also further underlines the central role of these cytokines in the pathogenesis of RA for the induction/regulation of disease-relevant molecules (30). Strong induction of IL-33 by IL-1β is in good agreement with previously published data in CNS glial cultures showing that IL-1β induces IL-33 more strongly than pathogen-associated molecular patterns, e.g., dsRNA or LPS (4). In contrast, superconfluent HUVECs responded with a down-regulation of IL-33 protein following IL-1β or TNF-α stimulation (5). Therefore, the regulation of IL-33 by the pro-inflammatory cytokines IL-1β and TNF-α seems to be cell type- and/or pathway-specific.

This study shows for the first time that IL-33 is involved in the regulation of TNF-α-induced functions in RA-SFs. Overexpression of IL-33 enhanced the TNF-α-induced reduction of proliferation in RA-SFs. Interestingly, the TNF-α-mediated downregulation of proliferation is solely dependent on the p38 signal pathway in RA-SFs (25). This is in good agreement with the regulation of TNF-α-induced IL-33 synthesis via p38 (Fig. 2) suggesting a downregulation of RA-SFs proliferation by TNF-α via a p38-IL-33 axis. The TNF-α/IL-33-induced downregulation of RA-SFs proliferation is in sharp contrast with previous reports suggesting induction of SFs proliferation by TNF-α (31,32). This discrepancy may be explained by the usage of differentially purified RA-SFs (anti-CD14-purified cells in the present publication vs. cells purified by passaging) and differential usage of serum-free or serum-containing medium.

Overexpression of IL-33 further augmented TNF-α-induced pro-inflammatory and pro-destructive functions in RA-SFs. Although this result was generally confirmed by IL-33 silencing, TNF-α-induced pro-destructive mediators were less strongly downregulated (MMP-1, MMP-3, TIMP-1). The difference in the effects of IL-33 overexpression and silencing on the regulation of TNF-α-induced pro-destructive functions may be based on the involvement of additional signaling pathways, e.g. p38 and NFκB (25,33). In fact, the inhibition/silencing of one signaling molecule can increase the activity of other signaling pathways influencing cell functions (34).

The present data indicate that IL-33 has an enhancing effect on TNF-α-induced pro-inflammatory and pro-destructive functions in RA-SFs. This is in apparent contrast to the initially proposed function of IL-33 as a transcriptional repressor (3). However, the evidence for this function of IL-33 is predominantly based on *in vitro* assays using reporter vectors exclusively driven by multiple galactose 4 binding sites (3,35). Also, there was no influence on the expression of selected genes by IL-33 overexpression or silencing in HUVECs (5,35). In addition, it has been proposed that IL-33 may function as a potentiator of gene expression by decreasing the local concentration of transcriptional repressors on specific promoters and thereby allowing activators to bind more efficiently (3). Thus, the precise transcriptional influence of IL-33 on the expression of individual genes in specific cell types will have to be further analyzed.

In the present study, overexpression and silencing of IL-33 influenced mRNA and protein expression of selected target genes to a comparable degree. This indicates that in RA-SFs IL-33 exerts its enhancing influence at the transcriptional level, either exclusively or in combination with other mechanisms. This is supported by recent data showing that a short motif of IL-33 binds with to the acidic pocket formed by the histone H2A-H2B dimer at the surface of the nucleosome, a region important for chromatin compaction and subsequent transcriptional activity (35) . However, the chromatin-binding motif of IL-33 induced a higher order structure of chromatin and mutations of the motif reduced its transcriptional repressor properties. It therefore remains the subject of future studies how the differential influence of IL-33 on gene transcription can be mechanistically explained.

The regulation of pro-inflammatory mediators by nuclear IL-33 is in good agreement with previously published data using inflammatory cells. Stimulation of these cells with IL-33 induced the synthesis of several pro-inflammatory mediators, e.g., IL-6, IL-8, and MCP-1 (7,36,37). However, in contrast to RA-SFs, these cells express ST2 and therefore responded to external IL-33 stimulation (37,38). Thus, pro-inflammatory mediators may be enhanced by nuclear IL-33 in cells not expressing the IL-33 receptor ST2.

Interestingly, lentiviral IL-33 overexpression enhanced IL-33 mRNA and protein expression only in TNF-α-stimulated RA-SFs, pointing to a stabilization of IL-33 by TNF-α. A similar effect has been reported for another member of the IL-1 family, IL-1F7b (39). In agreement with our observation for IL-33, IL-1F7b overexpressing RAW264.7 cells showed high intracellular IL-1F7b level only after LPS stimulation. Therefore, the mRNA of different members of the IL-1 family may be stabilized by pro-inflammatory stimuli, resulting in an increased protein synthesis.

The present study identifies IL-33 as a critical regulator of TNF-α-induced pro-inflammatory and pro-destructive functions in RA-SFs (likely at the transcriptional level) and raises interesting questions concerning cell type- or gene-specific effects and/or the exact molecular mechanism of gene regulation.

## Figures and Tables

**Table I:** Primer sequences and annealing temperatures used in RT-PCR.

Gene		Primer sequence	Annealing temperature, °C	Melting temperature, °C
Aldolase	Sense	5-tcatcctcttccatgagacactct-3	58	82
	Antisense	5-attctgctggcagatactggcataa-3		
IL-33	Sense	5-cacccctcaaatgaatcagg-3	60	84
	Antisense	5-ggagctccacagagtgttcc-3		
IL-6	Sense	5-atgaactccttctccacaagcg-3	62	84
	Antisense	5-ctcctttctcagggctgag-3		
IL-8	Sense	5-gccaagagaatatccgaact-3	60	78
	Antisense	5-aggcacagtggaacaaggacttgt-3		
MCP-1	Sense	5-cagccagatgcaatcaatgcc-3	60	82
	Antisense	5-tggaatcctgaacccacttct-3		
MMP-1	Sense	5-gacctggaggaaatcttgc-3	58	81
	Antisense	5-gttagcttactgtcacacgc-3		
MMP-3	Sense	5-gaacaatggacaaaggatacaaca-3	58	81
	Antisense	5-aagattgatgctgtttttgaagaa-3		
TIMP-1	Sense	5-cttctggcatcctgttgttg-3	60	82
	Antisense	5-agaaggccgtctgtgggt-3		

## Abbreviations

RA: rheumatoid arthritis
RA-SFs: RA synovial fibroblasts
OA: osteoarthritis
MCP-1: monocyte chemotactic protein-1
MMP: matrix metalloproteinase
